# Isolation of antibiotic-resistant strains of *Staphylococcus aureus* from raw milk produced by dairy cows with subclinical bovine mastitis

**DOI:** 10.5455/javar.2025.l892

**Published:** 2025-03-24

**Authors:** Saima Batool, Zubia Masood, Asim Ullah, Wali Khan, Mourad Ben Said, Hanène Belkahia, Alaa Bassuny Ismael, Ayman A. Swelum

**Affiliations:** 1 Department of Wildlife and Ecology, University of Veterinary and Animal Sciences (UVAS), Lahore, Pakistan; 2 Department of Zoology, Sardar Bahadur Khan Women’s University, Quetta, Pakistan; 3 Department of Fisheries and Aquaculture, University of Veterinary and Animal Sciences (UVAS), Lahore, Pakistan; 4 Department of Zoology, University of Malakand, Chakdara Dir Lower, Khyber Pakhtunkhwa, Pakistan; 5 Laboratory of Microbiology, National School of Veterinary Medicine of Sidi Thabet, University of Manouba, Sidi Thabet, Tunisia; 6 Department of Basic Sciences, Higher Institute of Biotechnology of Sidi Thabet, University of Manouba, Sidi Thabet, Tunisia; 7 Department of Clinical Laboratory Sciences, College of Applied Medical Sciences, Taif University, Taif, Saudi Arabia; 8 Department of Theriogenology, Faculty of Veterinary Medicine, Zagazig University, Zagazig, Egypt

**Keywords:** *Staphylococcus aureus*, antibiotic resistance, cow milk, dairy farms, Lahore

## Abstract

**Objectives:**

The rise of antibiotic-resistant strains of *Staphylococcus aureus* in dairy milk products is a global concern, compromising treatment efficacy and highlighting the need for innovative solutions. Therefore, a study was conducted to isolate *S. aureus* strains (*N* = 21) from raw milk samples of cows infected with subclinical bovine mastitis. Additionally, the resistance of these strains against 12 different antibiotics was examined.

**Materials and Methods:**

Sixty raw cow milk samples, 20 from each of three separate dairy farms in Lahore city, were collected and screened for the presence of *S*.* aureus.* It was discovered that 70% of these milk samples were contaminated with this bacterium, indicating a widespread presence across the farms. Different isolation tests were employed in this study, including gram staining, capsule staining, catalase, mannitol salt fermentation, DNase, coagulase, and oxidase.

**Results:**

The obtained results revealed that the isolated strains of *S. aureus* showed % of their resistance against different antibiotics in the order of amoxicillin (85%), penicillin (71%), gentamicin (CN) (42%), carbenicillin and trimethoprim-sulfamethoxazole (33%), streptomycin, ciprofloxacin, and oxytetracycline (28%), cefotaxime (10%), and chloramphenicol (4%) in decreasing order, respectively. However, these strains showed no resistance against Bacitracin and Ampicillin.

**Conclusion:**

The existence of resistant strains of *S*.* aureus* has been attributed to various factors, such as poor milk hygiene, delayed milk transportation, subclinical bovine mastitis among dairy cows, and antibiotic-resistant genes. Thus, our present study will provide useful information about the resistant strains of *S*.* aureus*, which may transfer through cows into milk and then produce serious food-borne diseases in human beings. This study will be helpful to improve and control the quality of dairy products in Pakistan.

## Introduction

Pakistan is the largest agriculture and livestock-producing country in the world because its dairy and livestock industries play a key role in its economy. Pakistan stands 5th with the production of 36.2 million tons of milk annually in the world. However, the milk or dairy industries of Pakistan sometimes face many problems, including unhealthy animals and infections due to the inattention of workers or milkmen that later may affect the quality of dairy products, which causes certain serious or minor infections in humans upon their consumption. Between 1996 and 2006, the distribution of livestock in Pakistan revealed that Punjab and Sindh provinces harbored a significant majority, with 72.4% of cattle and 92% of buffalo populations. Over the same period, milk production from cows and buffaloes increased by 35.6%, with an annual growth rate of 3.6%. This growth, particularly in cow milk production, resulted in cow milk’s share of total production rising from 33.1% to 34.7%. This increase can be attributed to the expansion of large cattle farms in Pakistan. In Punjab, the average yield of cow milk per animal/day is 6.31 L, and a total of 25,580,103 L of cow milk is produced per day [1].

*Staphylococcus aureus, *increasingly important as a major pathogen, is rising in significance due to its increasing resistance against antibiotics. It can affect most mammals, including humans. *Staphylococcus aureus* can be transmitted from animals as well as from one person to another through air droplets or aerosols suspended in the air by coughing or sneezing or direct contact with an object containing bacteria or through the bite of an infected animal. Approximately 30% of healthy humans harbor *S*. *aureus* in their noses, the back of their throats, and their skin [2]. It is obvious that in *S*.* aureus, *the most common antibiotic resistance was associated with its plasmids. The genetics of this bacterium are essentially similar to the genetics of *Escherichia coli*. Its resistance to antibiotics has increased physicians’ concern because 60% and 90% of *S*.* aureus *strains showed resistance against penicillin. Therefore, the use of antibiotics for its treatment often proves ineffective and exacerbates the issue of antibiotic resistance [3].

Milk, a vital staple in our diet, is consumed both fresh and as the base for various dairy products. Bovine mastitis, an inflammation of the mammary gland, occasionally results in systemic infection, significantly impacting animal welfare and milk quality. It is the most prevalent disease affecting high-yield dairy cows. The International Dairy Federation has recognized bovine mastitis as a critical issue in dairy animal pathology, emphasizing its significance. However, bovine mastitis, primarily caused by *S. aureus,* is a major challenge to the dairy sector. *Staphylococcus aureus* is responsible for 30%–40% of bovine mastitis cases globally, with Pakistan reporting a prevalence of 29%–57% in dairy cattle. Such a disease results in substantial economic losses, accounting for 17% of total animal disease-related costs in Pakistan and approximately $33B worldwide [4–7]. Contamination of milk due to mastitis can occur directly from infected udders or through improper handling, with bacterial loads reaching up to 10⁸ CFU/ml [8].

Current treatment methods for bovine mastitis, which rely on antibiotics, are often ineffective and contribute to the development of antibiotic-resistant strains. Therefore, this study aimed to identify the prevalence of *S. aureus* in raw milk from mastitis-affected cows in Lahore. Additionally, it also analyzed the antibiotic resistance profiles of the isolated strains of this bacterium to assess potential health risks and foodborne diseases among milk consumers.

## Materials and Methods

### Ethical approval

The study protocol and ethical considerations for this research have received approval from the Ethical Committee of the University of Veterinary and Animal Sciences (UVAS) in Lahore, under approval number FBS-QAU-90. A consent form was signed by the owners of dairy farms before sampling to get their approval for milk sampling from infected cows.

### Study area

Lahore is the second biggest city in Pakistan and is highly inhabited by 13 million people. It is the capital of Punjab province and is situated at 31°32’59” North and 74°20’37” East, and its total area is about 684 sq. meters. It is situated in the northeast of Pakistan, close to the border of India. The average temperature ranges from 21°C (December 2023) to 40°C (June 2023).

### Study design

**Figure 1.**
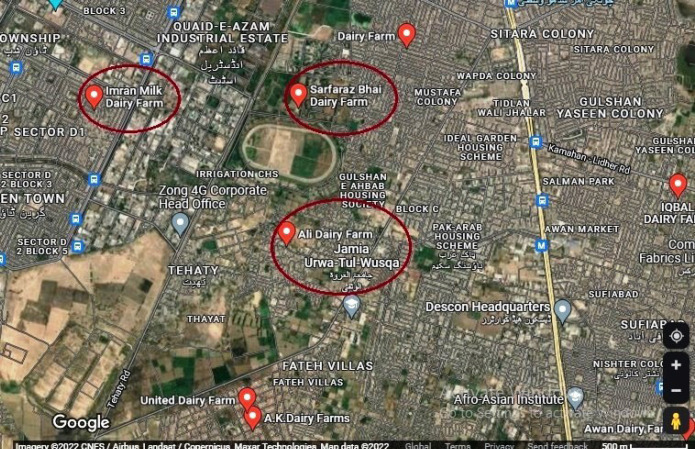
Google map of sampling sites in Lahore city of Pakistan.

During the period from July to December 2021, a total of 60 raw milk samples (20 samples from each dairy farm) of cows particularly infected with subclinical bovine mastitis were collected from three dairy farms in Lahore city of Pakistan, as shown in Figure 1, including, i.e., Ali Dairy Farms (SA1 = 20), Imran Milk Dairy (SA2 = 20), and Sarfaraz Bhai Dairy Farm (SA3 = 20), Lahore, for isolating and identifying 21 strains of *S. aureus* present in raw milk gathered from bovine mastitis cows and estimating their resistance against selected antibiotics.

### Sample size and collection

Sixty raw milk samples (*N* = 60) were gathered from infected cows with subclinical bovine mastitis present in three different dairy farms in Lahore. 10 ml milk samples were taken from each infected cow, placed in sterilized bottles, and immediately transferred to the QOL/WTO (Quality Operation Laboratory) of UVAS, as shown in Figure 1.

### Bacteriological isolation and identification

Milk samples were spread on the Mannitol salt agar and placed at 37°C for 24 h in an incubator to obtain isolated colonies. After incubation, size, shape, margins, pigmentation, elevation, and opacity are observed with the naked eye. The colonies were preliminarily identified through staining reactions with Gram stain and capsule staining. Colonies that grew on MSA were subcultured on nutrient media, and the cultures were preserved and maintained for isolates’ characterization. Oxidase tests, tube coagulase tests, and DNase tests were conducted on samples identified as positive for coagulase-positive *S*.* aureus* following the methods outlined by Girmay et al. [9].

### Determination of antibiotic vulnerability in S. aureus isolates

After confirming the presence of *S*.* aureus,* the effectiveness of antibiotics was assessed using the Kirby-Bauer (K-B) test, a standard method endorsed by the NCCLS (National Committee for Clinical Laboratory Standards) for antibiotic vulnerability testing, as described by Jackie-Reynolds [10] and Zhang et al. [11]. The K-B test, also known as the disc diffusion test, helps determine the resistance or sensitivity of bacteria to specific antibiotics, aiding in patient treatment decisions. The procedure involved swabbing the bacterium onto agar. The test procedure involves swabbing the bacterium onto agar, followed by placing antibiotic discs on top. As the antibiotics diffuse into the agar, their concentration decreases, creating varying levels of inhibition around the discs. If the organism is susceptible to the antibiotic, there will be a clear zone of inhibition where bacterial growth is inhibited. The size of these zones is measured and interpreted using standardized charts to determine bacterial sensitivity, resistance, or intermediate status. Many charts also provide the minimal inhibitory concentration (MIC) for each drug, offering more precise information on effective dosages. The Mueller-Hinton medium, a protein-rich agar, supports bacterial growth. Bacterial colonies are collected with sterile swabs and spread onto Muller Hinton plates to cover the entire surface. Antibiotic discs are then placed on the plates using sterile forceps, and the plates are incubated at 37°C for 24 h. Results were interpreted based on the MIC.

### Statistical analysis

All statistical analysis of data was performed by using MS Excel 2010 software.

## Results

### Identifying Staphylococcus species in cow-milk samples through cultural characteristics analysis

**Figure 2.**
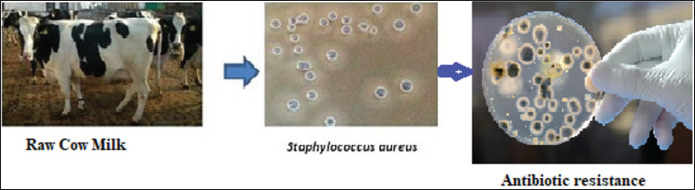
Isolation of *S. aureus* from raw cow milk and disc diffusion test of the bacteria for assessing antibiotic resistance patterns.

Sixty raw cow milk samples were obtained from infected cows with clinical and subclinical bovine mastitis present in three different dairy farms in Lahore. Then these milk samples were screened for identifying the occurrence of *S*.* aureus*. Out of these 60 raw milk samples, *S*.* aureus* strains were identified and present in only 42 raw milk samples (70%), while the remaining 18 samples (30%) were contaminated with some other bacterium species (Table 1). The presence of *S. aureus* in raw milk shows its poor quality, which can cause food-borne illnesses in consumers. Isolated *S*.* aureus* strains from milk samples were designated as SB1, SB2, SB3, SB4, SB5, SB6, SB7, SB8, SB9, SB10, SB11, SB12, SB13, SB14, SB15, SB16, SB17, SB18, SB19, SB20, and SB21. These isolates were differentiated into resistant, intermediate, and susceptible to antibiotics. The isolated strains of *S. aureus* showed their resistance against different antibiotics due to the presence of antibiotic-resistant genes (Fig. 2).

### Morphological and biochemical characterization

Golden yellow pigmented round colonies of convex shape appeared. Bacteria showed a purple color and are in a grape-like structure.* Staphylococcus aureus* is a gram-positive bacterium that showed a faint blue halo capsule around the purple cell as a result of capsule staining and showed positive results of catalase, mannitol salt fermentation, DNase, and coagulase but was oxidase negative.

### Analysis of zone of inhibition (in mm) by using the Kirby-Bauer method

**Figure 3.**
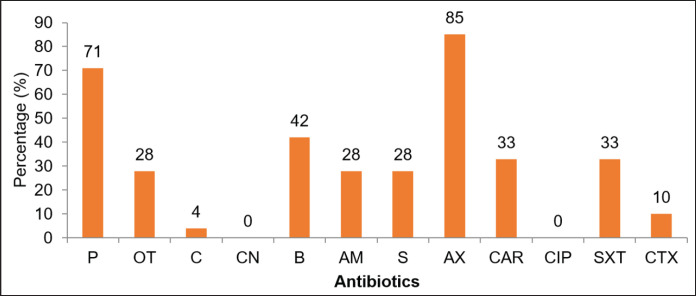
Antibiotic resistance of *S. aureus* against different antibiotics.

The Zone of Inhibition (in mm) in the Kirby-Bauer antibiotic susceptibility test indicates the effectiveness of an antibiotic against a particular bacterial strain. A larger zone suggests that the antibiotic is more effective in inhibiting the growth of the bacteria being tested. The MIC (minimum inhibitory concentration) that inhibits bacterial growth was also determined by using the Kirby-Bauer (K-B) method. This procedure determines the concentration of an antibiotic required to inhibit pathogen growth, providing insight into the effective dosage for controlling the infection in patients. For each antibiotic, 21 strains of *S*.* aureus* were differentiated into three categories, i.e., resistant, intermediate, and susceptible, according to their antibiotic potential, as shown in Table 2. The antimicrobial effect of each antibiotic depends on different factors such as (1) the severity of the infection of the animal from which milk was obtained and (2) the concentration of the antibiotic’s discs. The range of diameter of the zone of inhibition (ZOI) ranged from 8 to 20 mm. Some *S*.* aureus* strains showed resistance, while others had more susceptibility to antibiotics. The concentration of the antibiotic disc also had a great effect on MIC. A high concentration of dose had a significant effect on microbial growth, while a low dose had a low effect. The average value of MIC for the selected 12 antibiotics was also shown by 21 strains of *S*.* aureus *ranging from 11 to 25 mm, as shown in Table 3. *Staphylococcus aureus* strains indicated the highest resistance to both Amoxicillin and penicillin, which was 85% and 71%, respectively (Fig. 3), while showing no resistant effect against ciprofloxacin and gentamicin but revealing high susceptibility to these two antibiotics*. Staphylococcus aureus* resistivity for other antibiotics was found in order of Bacitracin 42%, Carbenicillin 33%, Trimethoprim-Sulphamethoxazole 33%, Ampicillin 28%, Oxytetracycline 28%, Streptomycin 28%, Cefotaxime 25%, and for Chloramphenicol 4%. Among them, penicillin shows the lowest diameter of MIC, which is 11 mm, whereas, cefotaxime shows the highest diameter of MIC, which is 25 mm.

**Table 1.** Collection of milk samples from three different Lahore dairy farms.

Sl No.	Sampling area	Sampling abbreviations/codes	No. of milk samples	No. of milk samples contaminated with bacteria isolates
1	Ali Dairy Farms Lahore	SA1	20	17^a^
2	Imran Milk Dairy Farm Lahore	SA2	20	13^b^
3	Sarfaraz Bhai Dairy Farm Lahore	SA3	20	12^c^
		Total	N = 60	42

Note: Superscript shows the highest and lowest values.

## Discussion

Bacteria play a crucial role in developing certain medicines used in clinical practice. Trabectedin, an anti-cancer drug derived from tunicates, is produced by their symbiotic bacteria. Similarly, many antibiotics, immunosuppressive drugs, and anticancer medications are derived from bacterial products. However, isolating antibiotics from the same environment where pathogenic strains reside can lead to resistance to naturally produced antibiotics [12,13]. *Staphylococcus aureus*, a major bacterium causing bovine mastitis, leads to significant economic losses in Pakistan’s dairy industry due to limited knowledge of its genetic diversity and antimicrobial resistance genes. This study highlights that initially, milk drawn from healthy animals may be bacteria-free but later becomes contaminated, either from the hands of milkmen or the udders of animals harboring microorganisms such as *Corynebacteria, Coliforms, Klebsiella,* *Streptococci, Staphylococci, *and *Salmonellae*. Dirty teats contaminated with dung and mud serve as sources of bacteria for milk contamination. Additionally, utensils used for milk storage and transportation also contribute to bacterial presence. However, the primary source of contamination often stems from adding contaminated water to milk to increase its volume. These findings indicate that raw milk undergoes unhygienic conditions during transportation, leading to significant contamination over time due to high temperatures, which promote bacterial proliferation. Montelongo et al. [14] noted that the genome of *Staphylococcus *contains a diverse array of genes, a significant portion of which are obtained through lateral gene transfer. Many antibiotic resistance genes are found on plasmids or other mobile genetic elements. The impressive capacity of *S. aureus* to acquire advantageous genes from diverse sources is underscored by the intricate nature of its genome and the clear evidence of lateral gene transfer.

Several studies, including those by Onanuga et al. [15], have noted significant resistance in *S. aureus* against various antibiotics. They found high resistance rates to ampicillin (91.7%), clindamycin (78.3%), cephalexin (75%), methicillin (71.7%), and vancomycin (68.3%) while showing very low resistance to gentamicin (3.3%), ciprofloxacin (3.3%), ofloxacin (3.3%), sparfloxacin (3.3%), and pefloxacin (10.0%). Another study by Ikeagwu et al. [16] indicated that *S. aureus* exhibited the highest sensitivity to Ofloxacin (65%) and the least to Co-trimoxazole (6%). Additionally, Gali et al. [17] discovered vancomycin-resistant *S*.* aureus* (VRSA) strains in milk samples in Nigeria, with 5.4% showing resistance. Notably, pasteurization and fermentation seemed to reduce the pathogen’s occurrence, as VRSA was absent in yogurt and less prevalent in pasteurized milk compared to raw milk. The VRSA strains exhibited high resistance to penicillin (100%), tetracycline (85%), amoxicillin (65%), methicillin (40%), and oxacillin (40%), but were sensitive to amikacin (5%) and sulfamethoxazole/trimethoprim (10%). Similarly, a study by Jahan et al. [5] revealed high resistance of *S. aureus* in raw milk of cows to amoxicillin (100%), penicillin (100%), and erythromycin (75%), while being sensitive to oxacillin (100%), cloxacillin (100%), neomycin (100%), and ciprofloxacin (83.33%). In our current work, *S. aureus *strains demonstrated the highest resistance to amoxicillin (85%) and penicillin (71%), whereas ciprofloxacin and gentamicin showed no resistance. Tanzin et al. [18] observed that both *S. aureus* and *Escherichia coli *are antibiotic-resistant strains found in milk samples of cattle and buffaloes in Bangladesh and can be controlled in the case of bovine mastitis by using antibiotics like ciprofloxacin and levofloxacin that showed low resistance rates. Additionally, Naushad et al. [19] studies highlighted the importance of antibiotic choice in controlling antibiotic-resistant strains in milk samples, with antibiotics like ciprofloxacin and levofloxacin showing low resistance rates. Furthermore, genetic diversity among *S. aureus* isolates from bovine milk revealed high resistance against beta-lactams and sulfonamides but low resistance against other antibiotics. Finally, Hassani et al. [20] investigations in Iran’s northwest regions found antibiotic resistance genes, such as *bla*_TEM_ and *bla*_SHV_ in *E*.* coli* and *Salmonella* species, and *mec*_A_ and *bla*_Z_ in *S. aureus* isolates, contributing to high rates of antibiotic resistance against amoxicillin, penicillin, and cephalexin, respectively.

**Table 2.** Determination of the diameter of the ZOI of* Staphylococcus aureus* isolated from cow milk.

S. No.	Antibiotics	Abbreviations	Disc Conc.	Diameter of Zone of inhibition (ZOI) in mm		
				Resistant	Intermediate	Susceptible
1.	Penicillin	P	10IU	≤ 20**^a^**	21–28**^a^**	≥ 29**^a^**
2.	Oxytetracycline	OT	30 µg	≤ 14^e^	15–18^d^	≥19^e^
3.	Chloramphenicol	C	30 µg	≤ 12^f^	13–17^e^	≥ 18^f^
4.	Gentamycin	CN	10 µg	≤ 12^f^	13–14^f^	≥ 15^h^
5.	Bacitracin	B	10U	≤ 8**^i^**	9–11^j^	≥ 13**^j^**
6.	Ampicillin	AM	10 mcg	≤ 11^g^	12–13^h^	≥ 14^i^
7.	Streptomycin	S	10 µg	≤ 11^g^	12–14^g^	≥ 15^h^
8.	Amoxicillin	AX	25 mcg	≤ 19^b^	NIL	≥ 20^d^
9.	Carbenicillin	CAR	100 µg	≤ 17^c^	18–22^b^	≥ 23^b^
10.	Ciprofloxacin	CIP	5 µg	≤ 15^d^	16–20^c^	≥ 21^c^
11.	Trimethoprim-Sulphamethoxazole	SXT	25 µg	≤ 10^h^	11–15^i^	≥ 16^g^
12.	Cefotaxime	CTX	30 µg	≤ 14^e^	15–17^d^	≥ 18^f^

Note: Superscript shows the highest and lowest ZOI values.

**Table 3.** Shows of antibiotic resistance potential of *Staphylococcus aureus* strains against twelve selected antibiotics.

Sr. no.	Antibiotics	Disc. Conc.	No. of strains of *S. aureus*			Antibiotic resistance (%)	Average value of MIC (mm)
			Resistant no. (%)	Intermediate no. (%)	Susceptible no. (%)		
1.	Penicillin	10IU	15 (71.4)	2 (9.5)	4 (19.04)	71.0^b^	11.0^i^
2.	Oxytetracycline	30 µg	6 (28.6)	0	15 (71.4)	28.0^e^	19.0^e^
3.	Chloramphenicol	30 µg	1 (4.8)	5 (23.8)	15 (71.4)	4.0^g^	22.0^c^
4.	Gentamycin	10 µg	0	2 (9.5)	19 (90.5)	0.0^h^	19.0^e^
5.	Bacitracin	10U	9 (4.29)	3 (14.3)	9 (42.9)	42.0^c^	11.0^i^
6.	Ampicillin	10 mcg	6 (28.6)	3 (14.3)	12 (57.1)	28.0^e^	16.0^f^
7.	Streptomycin	10 µg	6 (28.6)	4 (19.0)	11 (52.4)	28.0^e^	16.0^f^
8.	Amoxicillin	25 mcg	18 (85.7)	0	3 (14.3)	85.0^a^	14.0^h^
9.	Carbenicillin	100 µg	7 (33.3)	6 (28.6)	8 (38.1)	33.0^d^	20.0^d^
10.	Ciprofloxacin	5 µg	0	1 (4.8)	20 (95.2)	0.0^h^	24.0^b^
11.	Trimethoprim- Sulphamethoxazole	25 µg	7 (33.3)	2 (9.5)	12 (57.1)	33.0^d^	15.0^g^
12.	Cefotaxime	30 µg	2 (9.5)	1 (4.8)	18 (85.7)	10.0^f^	25.0^a^

Note: MIC = minimum inhibitory concentration. Superscript shows the highest and lowest values.

Over the past two decades, 75% of infectious diseases in humans have been traced back to animals, either directly or indirectly. *Staphylococcus aureus,* a global pathogenic strain, poses significant health risks, particularly through dairy products. While milk offers essential nutrients, improper handling during production and distribution can lead to food-borne illnesses, as it provides an ideal environment for harmful pathogens to thrive. *Staphylococcus *bacteria, including *S*.* aureus*, are commonly found in animals and humans, causing a range of ailments from mild skin infections to severe toxic shock syndrome and food poisoning. The emergence of antibiotic-resistant strains, notably methicillin-resistant *S*.* aureus*, presents a considerable challenge for healthcare and dairy industries. These resistant strains, including vancomycin-resistant *S*.* aureus*, pose therapeutic challenges and health risks, with milk being reported as a potential source of contamination [21]. Proper sanitation and hygiene practices are crucial in mitigating the spread of antibiotic-resistant bacteria through dairy products, safeguarding public health [9].

According to Schneider [22], antibiotic resistance has emerged as a significant challenge for public health and society at large. Despite the introduction of new antibiotics to the market, they too eventually encounter the rise of pathogenic resistance. The rapid development of resistance appears to be closely tied to the microbial origin and function of these antibiotics in nature. Most clinically used antibiotics are derived from natural microbial products, but the presence of antimicrobial resistance genes in their environment makes it easy for pathogens to acquire them through horizontal gene transfer. Janeoo et al. [23] have observed that drug-resistant bacterial pathogens are now causing major health issues affecting a significant portion of the global population. Therefore, there is an urgent need to discover effective antibacterial drugs against specific drug-resistant bacterial strains for healthcare systems worldwide.

## Conclusion

The raw cow milk used in Lahore City was contaminated with certain types of toxigenic or multidrug-resistant *S*.* aureus* strains that cause serious infections in consumers. Milk is a major source of food taken by human beings, and contaminated milk can cause various problems. Improving hygienic conditions and careful handling of cows during milking can effectively treat these issues. Thus, this study contributes to implementing suitable control measures to reduce contaminants and the spread of pathogenic bacterial strains in humans. We could further investigate the molecular characterization of the milk-isolated bacteria and the bacterial count per milliliter of milk.

## List of abbreviations

C, celsius; CFU, colony-forming unit; DNase, Deoxyribonuclease; h, hour; K-B, Kirby-Bauer; ml, milliliter; MIC, Minimal Inhibitory Concentration; mm, millimeter; NCCLS, National Committee for Clinical Laboratory Standards; VRSA, Vancomycin-resistant *Staphylococcus aureus*.
